# Platelet count in patients with severe coronavirus disease 2019

**DOI:** 10.17179/excli2020-3092

**Published:** 2021-01-04

**Authors:** Tomoyuki Kawada

**Affiliations:** 1 Department of Hygiene and Public Health, Nippon Medical School, 1-1-5 Sendagi, Bunkyo-Ku, Tokyo 113-8602, Japan

## ⁯

***Dear Editor,***

Recently, a majority of patients with severe coronavirus disease (COVID-19) have presented with thrombocytopenia. Figliozzi et al. (2020[1]) conducted a meta-analysis to clarify predictors of mortality in COVID-19. The following pooled odds ratios (OR) (95 % confidence intervals [CIs]) with respect to mortality were found: 3.61 (1.934-6.73) for steroid therapy, 3.15 (2.26-4.41) for a history of cardiovascular disease, 10.58 (5.00-22.40) for acute cardiac injury, 5.13 (1.78-14.83) for acute kidney injury, 4.8 (2.034-11.31) for increased procalcitonin, 3.7 (1.74-7.89) for increased D-dimer, and 6.23 (1.031-37.67) for thrombocytopenia. Additionally, the pooled ORs of advanced age, male sex, cardiovascular comorbidities, acute cardiac or kidney injury, lymphocytopenia, and D-dimer levels for in-hospital mortality also significantly increased. Similarly, Liu et al. (2020[4]) reported that the adjusted hazard ratio (95 % CI) of an increment of per 50 x 10^9^/L in the platelet count for mortality was 0.60 (0.43-0.84). They monitored changes in the platelet count during treatments and whether maintaining a normal platelet count was related to good prognosis. A meta-analysis conducted by Lippi et al. (2020[3]) revealed a pooled OR (95 % CI) of 5.1 (1.8-14.6) for thrombocytopenia with respect to mortality in patients with severe COVID-19.

However, some reports present an inverse association. Ibañez et al. (2020[2]) reported that prothrombin time, fibrinogen, and platelet levels were not reduced, as evidenced by low scores of disseminated intravascular coagulation (DIC) and sepsis-induced coagulopathy (SIC) on intensive care unit admission. Qu et al. (2020[5]) described the baseline platelet count, platelet-to-lymphocyte ratio (PLR), and their trends during treatment in 30 hospitalized COVID-19 patients. The PLR value at the peak platelet count during treatment was an independent influencing factor in severe patients. Specifically, PLR and the platelet count positively related to the average hospitalization period and could also predict prognosis of the disease. The dynamic changes in these parameters were speculated to be a cytokine storm. This is because these are recommended as the new indicators for monitoring COVID-19 patients. The mechanism for elevating the platelet count in patients with severe COVID-19 should be evaluated by considering subsequent progression of DIC and SIC.

Although two meta-analyses presented that thrombocytopenia reflected poor prognosis in patients with COVID-19 (Figliozzi et al., 2020[1]; Lippi et al., 2020[3]), the pooled OR had a wide range of CI. Nonetheless, continuous surveys are needed to confirm the association by excluding heterogeneity in meta-analyses.

## Conflict of interest

The author declares no conflict of interest.
